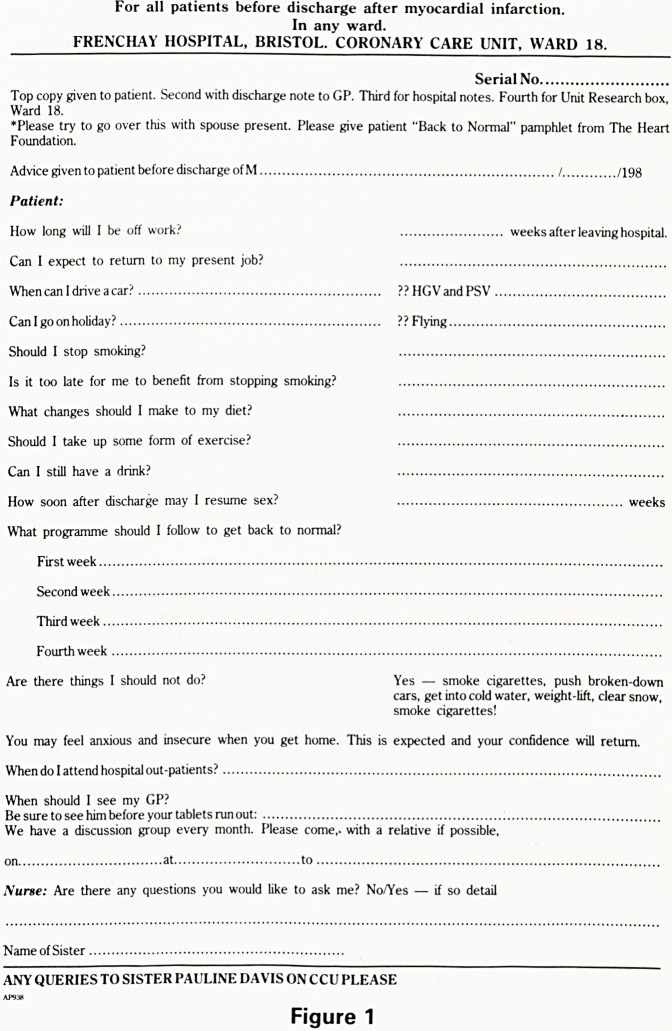# Assessment of Patient's Opinions on Personalised Advice Sheet Given before They Leave the Ward after a Myocardial Infarction

**Published:** 1986-10

**Authors:** Christopher Burns-Cox, Pauline Davis, Susan Teague

**Affiliations:** Consultant Physician and Nursing Sisters, Frenchay Hospital, Bristol; Consultant Physician and Nursing Sisters, Frenchay Hospital, Bristol; Consultant Physician and Nursing Sisters, Frenchay Hospital, Bristol

## Abstract

A personalised information and advice sheet for subjects with coronary artery disease was given to 120 consecutive patients leaving the Coronary Care Ward, Frenchay Hospital, after treatment for myocardial infarction. 5—10 months after discharge the 107 surviving patients were sent a letter enquiring about the benefits and weaknesses of this. Of 100 patients who replied, 97 said that the subject was fully covered and 98 that the sheet was helpful. Nevertheless, 14 found some topics difficult to understand and 13 found some worrying. The commoner problems were about fear and insecurity on leaving the ward, exactly how much physical exertion could be safely carried out, and how to cope with chest pains, particularly angina.


### Assessment of patients' opinions on personalised advice sheet given on leaving the ward after a myocardial infarction

**Christopher Burns-Cox, MD, FRCP, Pauline Davis, SRN, and Susan Teague, SRN**
Consultant Physician and Nursing Sisters, Frenchay Hospital, Bristol

### ABSTRACT

A personalised information and advice sheet for subjects with coronary artery disease was given to 120 consecutive patients leaving the Coronary Care Ward, Frenchay Hospital, after treatment for myocardial infarction. 5–10 months after discharge the 107 surviving patients were sent a letter enquiring about the benefits and weaknesses of this. Of 100 patients who replied, 97 said that the subject was fully covered and 98 that the sheet was helpful. Nevertheless, 14 found some topics difficult to understand and 13 found some worrying. The commoner problems were about fear and insecurity on leaving the ward, exactly how much physical exertion could be safely carried out, and how to cope with chest pains, particularly angina.

### INTRODUCTION

Both on the ward and after discharge after a myocardial infarction, patients and relatives have confused ideas on the advice they are given (1, 2, 3).

In the absence of a formal system, it is unlikely that many patients or relatives will be given adequate education and advice. Probably they will also not have enough time to voice their anxieties and fears. At present, general practitioners (GPs) are seldom told the exact advice given in hospital and this may lead to a confused patient and family. We felt the simplest way to ensure that the chief areas of concern were fully discussed before discharge was to use a form to be completed for each individual and given to him with a copy to the GP.

We have done this and now have looked at some aspects of what has happened.

### PATIENTS AND METHODS

120 successive patients treated for a myocardial infarction on the Coronary Care ward at Frenchay Hospital were given a personalised advice sheet (Figure 1). This was completed with the patient by the Senior House Officer or House Physician. Other copies went to the General Practitioner with the discharge note and to the hospital case notes. Five to ten months after discharge, a letter was sent with a stamped addressed envelope to each surviving patient with questions requiring yes/no answers, and also asking for comments and advice for future improvements.

### RESULTS

Of the 120 patients, 13 had died, 2 were untraceable, 1 had emigrated, and 4 failed to reply. Of the 100 responders, only 3 failed to answer every question and only 4 answers were not given.

**For all patients before discharge after myocardial infarction.**
**In any ward.**
**FRENCHAY HOSPITAL, BRISTOL. CORONARY CARE UNIT, WARD 18.**

**Serial No.........................**

Top copy given to patient. Second with discharge note to GP. Third for hospital notes. Fourth for Unit Research box, Ward 18.
*Please try to go over this with spouse present. Please give patient "Back to Normal" pamphlet from The Heart Foundation.

Advice given to patient before discharge of M ........................................ /........../198

***Patient:***

How long will I be off work? ...................... weeks after leaving hospital.

Can I expect to return to my present job? ........................................

When can I drive a car? ........................................ ?? HGV and PSV ........................................

Can I go on holiday? ........................................ ?? Flying ........................................

Should I stop smoking? ........................................

Is it too late for me to benefit from stopping smoking? ........................................

What changes should I make to my diet? ........................................

Should I take up some form of exercise? ........................................

Can I still have a drink? ........................................

How soon after discharge may I resume sex? ........................................ weeks

What programme should I follow to get back to normal?

First week ........................................

Second week ........................................

Third week ........................................

Fourth week ........................................

Are there things I should not do? Yes — smoke cigarettes, push broken-down cars, get into cold water, weight-lift, clear snow, smoke cigarettes!

You may feel anxious and insecure when you get home. This is expected and your confidence will return.

When do I attend hospital out-patients? ........................................

When should I see my GP?
Be sure to see him before your tablets run out: ........................................
We have a discussion group every month. Please come,. with a relative if possible, on............................at..........................to ........................................

***Nurse:*** Are there any questions you would like to ask me? No/Yes — if so detail

........................................

Name of Sister ........................................

**ANY QUERIES TO SISTER PAULINE DAVIS ON CCU PLEASE**
AP938

**Figure 1**

The answers to the questions were as follows:

| | | |
|---|---|---|
| Were the items on the advice sheet fully explained to you? | *Yes*: 97 | *No*: 3 |
| Did you find the advice sheet helpful? | *Yes*: 98 | *No*: 2 |
| Did you find any items difficult to understand? | *Yes*: 14 | *No*: 85 |
| Did you find any item worrying? | *Yes*: 13 | *No*: 87 |
| Were any topics left out you would like included? | *Yes*: 22 | *No*: 78 |
| Did you receive the British Heart Foundation pamphlet 'Back to Normal'? | *Yes*: 74 | *No*: 24 |
| Did you find it helpful? | *Yes*: 71 | *No*: 2 |
| Could it be improved? | *Yes*: 10 | *No*: 63 |
| Was there anything about your illness or hospital stay you think we could improve on? | *Yes*: 15 | *No*: 85 |

Patients' comments were put in 4 groups.

*(continued on page 119)*

### Assessment of patients' opinions *(continued from page 105)*

*Advice unclear*:
Normal life is contradicted by Do's and Don'ts.
How long shall I feel generally unwell?
At what times can patients do special things?
How far to go without overtaxing the heart?

*Advice omitted*:
Explaining angina and what brings it on.
What is a coronary bypass and when is it done?
What caused my heart attack?
Was my heart attack mild or serious?
Overcoming initial fear of small physical tasks.
Loss of confidence experienced on leaving the ward.
Indication of mental adjustment needed. This was the most difficult part of recovery.
Side effects of drugs prescribed.

*Practical comments on hospital stay*:
Call-bell and light control difficult to reach.
Food uninteresting and full of fat.
Would like BBC World Service on hospital radio at night.
Night nurses to chat to sleepless patients.

*Personal experiences mentioned*:
I found angina frightening.
It took me longer than advised to feel well enough to start work.
No special diet in hospital but told of one on discharge.
Needed more reassurance about twinges of anxiety pain and indigestion pain.
Fear of another attack is always present so how can one get back to normal?
Would feel more secure if given another hospital check-up at 6 months.
There were comments of praise and/or thanks for the help received from 24 patients.

### DISCUSSION

It is encouraging that 98% of patients found the sheet helpful and that 97% felt that the details had been adequately explained to them. The rush and pressures of the lives of house physicians and senior house officers is not conducive to careful prolonged discussion.

Topics such as angina, what to do if further chest pains and the feeling of insecurity on homegoing plainly need to be included in advice sheets in future. The items causing worry were those where worry for a while is inevitable: the increased risk of further infarction and sudden death cannot be explained away and the patient has either to practice denial or develop a philosophy which includes a degree of acceptance of death. In units such as ours, with five consultant physicians responsible for patients in the ward, it is dangerously confusing for nurses and patients if patients are advised and treated too differently. It is likely that a ward sister with extensive experience of coronary care is the ideal person to give detailed advice.

Staff responsible for the care of patients with myocardial infarction should have a rehabilitation policy. We would suggest that there are three phases for consideration.

Firstly, discussion and guidelines given to patient and family before discharge in one study, 47% of patients with their relatives failed to be taught about their illness (4). The GP should be told exactly what advice has been given.

Secondly, patient and family should be encouraged to attend a group discussion 3–6 weeks after infarction. This can reduce feelings of isolation in the patient, help spouses and give feedback to hospital staff (5, 6).

Thirdly, for selected patients with difficulties in returning to normal, graded physical training and teaching in stress management and relaxation should be available (7).

We would like to thank our medical and nursing colleagues for their co-operation, and Imperial Chemical Industries for printing the advice sheets.

### REFERENCES

1. RAHE R. R., SCALZI, C., SHINE, K. A teaching evaluation questionnaire for post myocardial infarction patients. Heart and Lung 1975; 4: 759.
2. MAYOU R., WILLIAMSON, B., FOSTER, A. ? and advice after myocardial infarction. Br Med J 1976; 1: 1577–1579.
3. DOMINION, J. AND DOBSON, M. Br Med J 1969; 2: 795.
4. JENKINS, B., KENT, S., MAYBERRY, J. F. and COLBOURNE, G. Patients' evaluation of a post-myocardial infarction teaching programme administered by nurses. Postgrad Med J 1984; 60: 108–110.
5. ROBINSON J. Group sessions direct rehabilitation after myocardial infarction. J Roy Coll Physicians 1983; 17: 213–216.
6. RAHE, R. H., O'NEIL, T., HAGAN, A. and ARTHUR, R. J. BMEF group therapy following myocardial infarction: ? follow up of a controlled trial. Int J Psychiat Med 1975; 6: 349–358.
7. LANGOSCH, W., SEER, P., BRODNER, L., LALLINKE, D., KULUK, B., HEIM, F. Behaviour therapy with coronary heart disease patients: results of a comparative study. J Psychosomato Res 1982; 26: 475–484.

## Figures and Tables

**Figure 1 f1:**